# Internationally standardized respiratory viral load testing with limited resources: A derivative‐of‐care calibration strategy for SARS‐CoV‐2

**DOI:** 10.1111/irv.13207

**Published:** 2024-01-23

**Authors:** Lili Tao, Allison Chan, Alex Maris, Jonathan E. Schmitz

**Affiliations:** ^1^ Department of Pathology, Microbiology, and Immunology Vanderbilt University Medical Center Nashville Tennessee USA; ^2^ Department of Urology Vanderbilt University Medical Center Nashville Tennessee USA; ^3^ Vanderbilt Institute for Infection, Immunology, and Inflammation Vanderbilt University Medical Center Nashville Tennessee USA

**Keywords:** International Units, SARS‐CoV‐2, standardization, viral load

## Abstract

**Introduction:**

SARS‐CoV‐2 has demonstrated that, in targeted circumstances, viral quantification within respiratory specimens can valuably inform patient management, as well as research. Nevertheless, the pandemic has illustrated concomitant challenges for obtaining high‐quality (and broadly comparable) respiratory viral loads. This includes a critical need for standardization and calibration, even though the necessary resources may not always be available for emergent pathogens and non‐bloodstream specimens.

**Methods:**

To these ends, we describe a novel strategy for implementing quantitative SARS‐CoV‐2 testing with International Unit‐based calibration. Earlier in the course of the pandemic—when analytic resources were far more limited—select residual SARS‐CoV‐2 positive specimens from routine care in our diagnostic laboratory were pooled to formulate a clinically realistic secondary standard of high volume and analyte concentration, which was cross‐calibrated to the primary SARS‐CoV‐2 standard of the World Health Organization.

**Results:**

The resultant calibrators were integrated into the original CDC RT‐qPCR assay for SARS‐CoV‐2, whose (now broadened) performance characteristics were defined to generate a test appropriate for both clinical and research use. This test allowed for the quantification of virus in respiratory specimens down to a validated lower limit of quantification of 10^3.4^ IU/ml.

**Conclusions:**

By self‐formulating calibrators from this derivative‐of‐care secondary standard, we successfully validated respiratory viral loads without the commercial availability (at that time) of quantitative assays or calibrators. As the SARS‐CoV‐2 pandemic continues to decline—and even beyond this pathogen—this strategy may be applicable for laboratories seeking to implement viral load testing for nontraditional specimen types despite limited resources.

## 1 INTRODUCTION

Diagnostic viral load measurements are an important aspect of care for many infections, with testing most often performed on plasma/serum or whole‐blood specimens. For instance, bloodstream viral quantification is used to monitor the progression and therapeutic response of HIV/AIDS,^1^ hepatitis B,^2^ and hepatitis C.^3^ Viral load testing is likewise common for human herpesviruses (EBV, CMV, HHV‐6) to evaluate for reactivated infection, especially in solid organ and stem cell transplant recipients.^4^, ^5^, ^6^ Various diagnostic platforms have been developed around quantitative testing needs, including products with formal regulatory approval (i.e., in vitro *diagnostic* status from the US Food and Drug Administration or European Community). Most such assays are based upon the real‐time polymerase chain reaction, targeting either DNA (qPCR) or RNA (RT‐qPCR) microbial targets.^7^ Other technologies for nucleic acid amplification tests (NAATs) are likewise conducive to quantification, including real‐time transcription‐mediated amplification (TMA)^8^ and digital droplet PCR (ddPCR).^9^

Beyond the bloodstream, these techniques can facilitate quantification of viruses (or nonviral pathogens) from other specimen types. Less common, but potentially relevant, clinical matrices include urine, stool, cerebrospinal fluid (CSF), and bronchoalveolar lavage (BAL) fluid.^10^, ^11^ The value of quantification in such cases is highly dependent on the specific pathogen, anatomic site, and clinical question. Nevertheless, in select circumstances, pathogen quantification can add value beyond just a result of *Detected* versus *Not Detected* (often referred to as a “categorical” or “semiquantitative” result). Examples include HIV quantification within CSF to diagnose neurocompartmentalized infection,^12^ quantification of BK virus within urine to differentiate viral hemorrhagic cystitis from nonspecific BK reactivation,^13^ and quantification of *Pneumocystis jerovici* within respiratory specimens to differentiate symptomatic infection from low‐level colonization.^14^

At the same time, although qPCR is not theoretically limited to any one pathogen or specimen type, implementing quantitative testing for atypical circumstances can be challenging. Preformulated commercial options may not be widely available, making it contingent upon individual laboratories to develop their own methodologies. In the United States, this implies local validation of a laboratory‐developed test (LDT) according to the performance characteristics stipulated by the Clinical Laboratory Improvement Amendments.^15^ In addition, although the C_t_‐values of qPCR create the potential for quantification, the technique itself does not generate an absolute viral load (VL). The latter requires the addition of relevant calibrators, which correlate empiric C_t_‐values with numeric concentrations. Without calibration, C_t_‐values only reflect a target's relative abundance for a given primer/probe set and testing system (inclusive of the extraction method, specific biochemical reagents, PCR instrumentation, and C_t_‐threshold settings). A set of calibrators, in turn, is typically derived from a *standard* for that pathogen, a certified reference material that carries a defined concentration and units.^16^, ^17^ Ideally, a set of molecular calibrators could demonstrate the following properties:

- They are traceable metrologically to some primary standard that can ensure comparability across laboratories. For some pathogens, a primary standard has been promulgated by the World Health Organization (WHO), defined by convention in International Units (IU).^18^
- Calibrators should reflect both the specific clinical matrix being tested (e.g., bloodstream vs. respiratory), as well as the state of the pathogen as it exists in the host (intact viral particles, as opposed to pre‐extracted viruses or contrived nucleic acids).
- Within an assay, calibrators are subject to the same extraction procedure, as well as all downstream methodologic steps, as actual patient specimens.
- Calibrators should reflect the range of concentrations encountered for that pathogen in the given specimen type.
- Although not an absolute requirement, cost‐favorability is an attractive feature for molecular reagents, as commercial standards/calibrators can be expensive.

Against this backdrop, the COVID‐19 pandemic has generated an unprecedented demand for molecular diagnostics over the past 3 years.^19^ NAATs have become the gold standard for detecting SARS‐CoV‐2, with testing most often performed on nasal or nasopharyngeal swabs inoculated into viral transport media (VTM).^20^, ^21^ Many NAATs for SARS‐CoV‐2 employ RT‐qPCR, whose logistical advantages include ease of multiplexing and the simultaneous generation and detection of amplicons. Within the United States, the Centers for Disease Control and Prevention (CDC) promulgated the first RT‐qPCR test for SARS‐CoV‐2 to be granted Emergency Use Authorization (EUA) by the FDA.^22^ This Taq‐man‐based “CDC assay” remained in prominent use for ~2 years in the United States, even with the subsequent authorization of numerous commercial platforms. A noted strength of the CDC assay was the flexible incorporation of its primer/probe sets with a number of validated extraction techniques, master mixes, and thermocycling instruments.

Despite employing RT‐qPCR, the CDC assay (as well as other NAATs for SARS‐CoV‐2) was developed and authorized as categorical/semiquantitative tests (*Detected*/*Not Detected*) without quantitative calibration.^22^ In general, additional validation steps are required to deploy an assay quantitatively, for reasons of both analytic rigor and regulatory compliance.^15^ Notably, one must establish the dynamic range of a quantitative test, for which the empiric read‐out varies predictably (typically linearly) with the concentration of analyte. Beyond assay validation, a fundamental consideration for SAR‐CoV‐2 is whether a quantitative VL adds value to a categorical/semiquantitative result. Indeed, for a majority of patients undergoing testing for this virus, the *Detected*/*Not Detected* result is the key piece of actionable data that guides clinical decision‐making.^23^ Additional VL data is not necessarily desirable if it does not alter management, and quantitative results can be more prone to misinterpretation by nonexperts. In this light, it is perhaps unsurprising that diagnostic NAATs for SARS‐CoV‐2 were promulgated without quantification.

Nevertheless, given the scale of the pandemic and the symptomatic diversity of COVID‐19, clinical scenarios can still arise in which respiratory VLs meaningfully guide care. Many such cases involve immunocompromised populations, who can experience prolonged illness with sustained viral replication. Here, serial VL measurements can track a patient's response to treatment or help differentiate prolonged infection from confounding phenomena.^24^ The latter include the possibility of SARS‐Cov‐2 reinfection, as well as the persistent detection of residual, low‐level viral RNA in the absence of viral replication.^25^ Moreover, the respiratory burden of SARS‐CoV‐2 can reflect a patient's capacity to transmit the pathogen, including potentially as a surrogate for replication‐competent virus.^26^, ^27^, ^28^ Studies have correlated a specimen's C_t_‐value with its ability to infect cells, with high C_t_‐values reflecting noninfectious viral remnants.^29^

Many diagnostic laboratories have grown accustomed to inquiries about releasing RT‐qPCR C_t_‐values for SARS‐CoV‐2 as part of the reported result. Even when clinically justified, however, such requests can be problematic. From a medicolegal perspective, a laboratory should not report a quantitative value on a test that has not been adequately validated for that purpose.^15^ Analytically, moreover, C_t_‐values can vary considerably on the same specimen when analyzed across different testing platforms. This critical nuance has been highlighted for SARS‐CoV‐2, including the risk of overinterpreting isolated C_t_‐values.^30^, ^31^, ^32^, ^33^ As discussed, absolute quantification (and comparability between platforms) requires the availability and validation of appropriate calibrators.

By late 2020, our clinical laboratory was facing demand for respiratory VLs (a small proportion of SARS‐CoV‐2 testing overall, but often entailing complex clinical scenarios). However, without any commercial quantitative assays with IVD status, this need could only be addressed through validation of a local method. To these ends, we attempted to build upon the CDC's original PCR assay. Specifically, we sought to validate and incorporate external calibrators into the existing protocol. But this task was not trivial, given inherent resource limitations. Commercial standards were not widely available that emulated native SARS‐CoV‐2, particularly at the high concentrations encountered in respiratory specimens. In this context, obtaining a high‐concentration viral standard to formulate calibrators would have necessitated in vitro SARS‐CoV‐2 culture, beyond the capacity of typical diagnostic laboratories. Even more fundamentally, the basic conventions used to quantify SARS‐CoV‐2 remained highly nonuniform, with scales that relied upon both biochemical units (e.g., copies/ml) and functional measures of infectivity (e.g., TCID_50_/ml).

Facing this logistical and analytic dilemma, we chose to leverage two resources still at our disposal. Although only available in small quantities, the WHO had recently promulgated the first international molecular standard for SARS‐CoV‐2 in standardized units of IU/ml.^34^ And although we could not report C_t_‐data from the original CDC assay, these values were available internally. Our laboratory was naturally aware of, and had massive access to, residual respiratory specimens with low C_t_‐values/high viral concentrations. Accordingly, numerous such specimens were pooled to create a high‐volume secondary standard, whose concentration was defined (in IU/ml) against the primary WHO standard. This secondary standard was serially diluted (in negative respiratory matrix) to generate a set of clinically realistic and fully extractable calibrators. These were incorporated into each iteration of original CDC assay and validated as a quantitative LDT appropriate for clinical deployment. Overall, this strategy represents an analytically rigorous—yet imminently practical—solution to a real‐world diagnostic problem, one that might be utilized beyond SARS‐CoV‐2 for laboratories facing similar resource limitations for quantitative assay development.

## 2 METHODS

### 2.1 FDA‐authorized testing for SARS‐CoV‐2

The work described here was performed within the Clinical Laboratory of Vanderbilt University Medical Center (VUMC), a CLIA/CAP‐accredited environment that provides inpatient and outpatient diagnostic testing for the affiliated academic health system. Throughout much of 2020 and into 2021, one of the primary molecular diagnostics employed here (and elsewhere) for SARS‐CoV‐2 was the CDC RT‐qPCR assay. This panel utilizes two primer/probe sets to amplify distinct regions of the viral nucleocapsid (N) gene, N1 and N2.^22^ As an internal control, the panel incorporates an additional primer/probe set for the human RNase P gene (RP). Of note, the assay allowed laboratories to implement these EUA‐status oligonucleotides (acquired here from Integrated DNA Technologies) with various equipment/reagents that may be available locally (i.e., different extraction kits, PCR master mixes, and thermocyclers). At VUMC, viral RNA was purified from VTM specimens via the Biomerieux eMAG platform. Amplification and detection were carried out in TaqPath 1‐Step CG master mix (Applied Biosystems) on the QuantStudio 7 Flex qPCR platform (Applied Biosystems, v1.3 software). The interpretation of positive/negative results was conducted according to the CDC product insert.^22^

Further molecular analyses were conducted on the Hologic Panther‐Fusion platform, an automated system that can perform RT‐qPCR on its modular Fusion component. An IVD‐status assay has been developed by Hologic for SARS‐CoV‐2 for the Fusion.^35^ C_t_‐values are not included as part of the reported clinical result, although they are available to the operator as raw data. The specific viral genomic targets of this assay remain proprietary. For the work here, calibrators (in VTM) were inoculated into commercial *specimen lysis tubes* (Hologic) and loaded onto the instrument for automated handling.

### 2.2 Acquiring primary/secondary quantitative standards

The First WHO International Standard for SARS‐CoV‐2 RNA was obtained from the National Institutes for Biological Standards and Control (UK), the material's exclusive distributor, defined at a concentration of 7.7‐log_10_ IU/ml (i.e., 10^^7.7^ = 5.01 × 10^7^ IU/ml). A secondary standard, in turn, was formulated locally with residual clinical samples from the VUMC Clinical Laboratory. The latter consisted of nasopharyngeal swabs inoculated into viral transport medium (Copan UTM‐RT), utilized after initial diagnostic testing for SARS‐CoV‐2 was complete. Specimens were selected if they demonstrated C_t_‐values of <17 for both the N1 and N2 targets of the CDC assay (not part of the clinically reportable result); these were pulled and frozen at −80°C within 24 h of the original analysis. No additional inclusion/exclusion criteria were applied for these specimens, other than selecting no more than one specimen from a given patient.

A total of 150 such specimens were collected over a 4‐week period in February/March 2021. They were thawed and combined at 1 ml/specimen (within a biological safety cabinet) to create a large‐volume pool. A corresponding pool was formulated from a commensurate number/volume of residual SARS‐CoV‐2 negative specimens, serving as the relevant clinical matrix for necessary dilutions. Positive and negative pools were re‐aliquoted and frozen for long‐term use.

### 2.3 Assigning a concentration to the secondary standard

The qPCR reactivity of the pooled secondary standard was correlated with that of the primary standard. Ten‐fold dilutions were made (within pooled negative matrix) of both the primary (0.2X–0.0002X) and secondary standards (1X–0.0001X). These were extracted like routine samples and analyzed together on the CDC assay (as described above). This protocol was repeated across 8 days, with duplicate technical measurements daily. Parallel‐line analysis (PLA 3.0, Stegman Systems) was applied to the resultant C_t_‐dataset to assign an IU‐based concentration to the secondary standard, for both N1 and N2 primer/probe sets. This material was diluted in a negative matrix to create a set of calibrators (10^8.5^–10^4.5^ IU/ml) to be extracted and included in the CDC assay.

### 2.4 Dynamic range validation of the quantitative assay

With the incorporation of calibrators (derived from the above secondary standard), the LLOQ of the modified CDC assay was determined as described by the Clinical and Laboratory Standards Institute (CLSI).^28^ Across four separate days and two operators, eight lots of low‐positive SARS‐CoV‐2 material were contrived by inoculating the primary WHO standard into eight VTM specimens from single SARS‐CoV‐2 negative patients. Each lot was formulated at the concentrations near/below that of the lowest calibrator; these validation specimens (three technical replicates for each lot/concentration) were extracted as routine patient samples and analyzed alongside all secondary calibrators. For each such specimen, a “calculated” VL was determined based on its observed C_t_‐value and linear regression analysis of the calibrators. This value was compared with its “defined” VL, known a priori from its dilution from the primary standard. At each formulated viral concentration, variance and bias across these measurements were used to assign total error (TE) according to root mean squared (RMS) and Westgard methods.^36^

## 3 RESULTS

### 3.1 Developing a quantitative standard and calibrators for SARS‐CoV‐2

**FIGURE 1:**
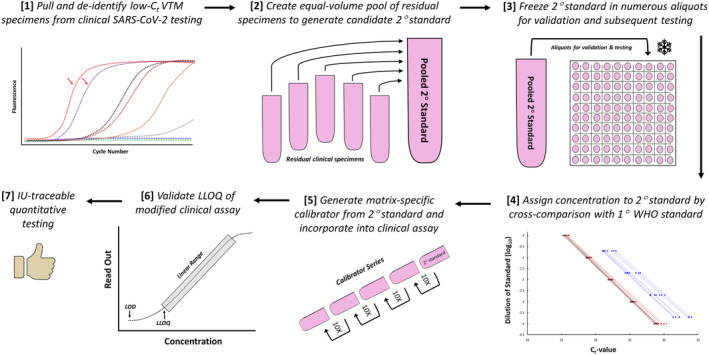
Schema for developing secondary standard/calibrators. Summarized here is the stepwise process that was employed for developing a derivative‐of‐care secondary standard, quantifying its concentration relative to the primary WHO standard, and utilizing the resultant calibrators to validate a quantitative assay for SARS‐CoV‐2 respiratory viral loads.

For our laboratory to develop quantitative SARS‐CoV‐2 testing, a quantitative standard was required. To these ends, the first WHO international standard for SARS‐CoV‐2 RNA had recently been promulgated, defined in units of IU/ml, to which secondary standards could be generated. With no viable commercial options, we took the novel approach of pooling residual nasopharyngeal VTM specimens—those with the lowest C_t_‐values—encountered during routine testing with the CDC assay. As summarized in Figure 1, this process generated a large volume of *normal* (i.e., averaged across many patients) clinical matrix with a high concentration of SARS‐CoV‐2. With this pool as a secondary standard, dilutions of it would possess the key features desired of molecular calibrators (as discussed in Section 1).

**FIGURE 2:**
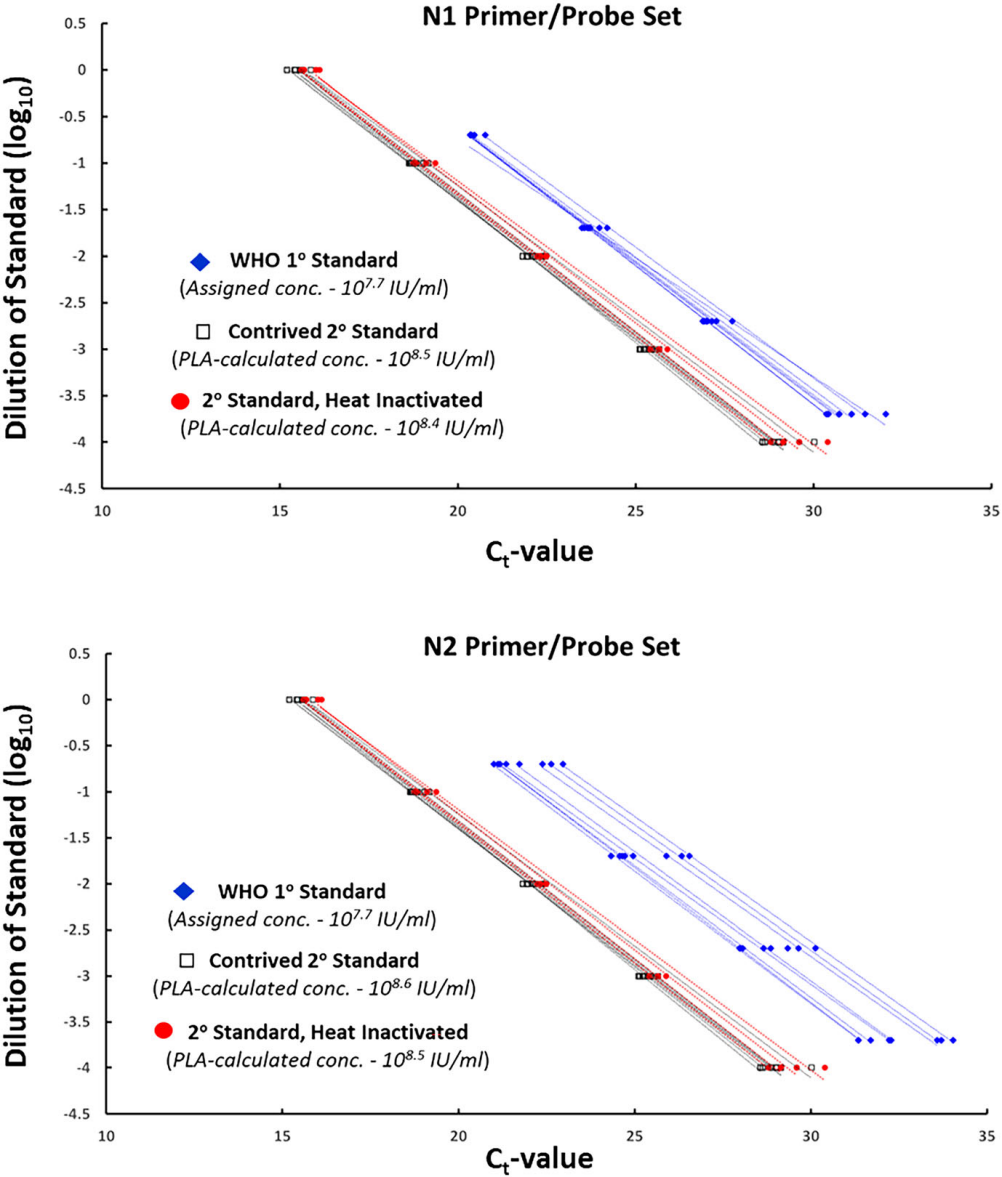
Linear RT‐qPCR response of primary and secondary standards. For both the N1 (top) and N2 (bottom) primer/probe sets, primary (WHO) and secondary (pooled clinical derivatives) standards for SARS‐CoV‐2 were diluted in negative matrix and subject to RT‐qPCR by the CDC assay. Heat‐inactivated secondary standard was also included. The C_t_‐versus‐dilution response is plotted here for each specimen and independent experimental replicate, together with in‐replicate linear regression trendlines (which universally demonstrated *R* ^2^ > 0.99). These data were employed to assign concentrations to the secondary standards, via parallel lines analysis.

We first sought to demonstrate the concentration‐versus‐C_t_ response within the CDC assay for both the WHO standard and the pooled secondary standard. When diluted up to four orders of magnitude from their original stocks (in pooled SARS‐CoV‐2 negative VTM), these standards demonstrated highly log‐linear performance. In each of eight independent replicates for the N1 and N2 targets, regression analysis uniformly yielded *R* ^2^ > 0.99 for both standards and primer/probe sets (Figure 2). In conjunction, we applied PLA to these datasets to calculate an IU‐based concentration for the secondary standard, according to WHO guidelines for preparing molecule reference materials.^37^ Based on the assigned concentration of the WHO standard (10^7.7^ IU/ml), PLA yielded a secondary standard concentration of 10^8.50^ IU/ml based on N1 (95% confidence interval: 10^8.47^–10^8.53^) and 10^8.56^ IU/ml based on N2 (95% confidence interval: 10^8.53^–10^8.59^). From these combined, a final concentration of 10^8.5^ IU/ml was assigned to the secondary standard for all subsequent work.

In parallel, we assessed the dose–response (dilution vs. C_t_‐value) of the secondary standard following heat inactivation of the virus. Aliquots of the secondary standard were treated for 15 min at 95°C prior to serial dilution in pooled negative VTM; specimen extraction and all subsequent steps of the CDC assay were performed as before. This deactivated pool likewise demonstrated a highly log‐linear relationship between relative concentration and C_t_‐value for both the N1 and N2 primer/probe sets (Figure 2). PLA yielded a virtually identical concentration to this deactivated pool as its non‐deactivated counterpart: N1‐based calculation = 10^8.44^ IU/ml (95% confidence interval: 10^8.42^–10^8.46^); N2‐based calculation = 10^8.45^ IU/ml (95% confidence interval: 10^8.39^–10^8.50^). These analyses demonstrate that if a laboratory were inclined to utilize deactivated residual specimens to create a local standard—for instance, out of biosafety motivations—it is a viable alternative.

### 3.2 Validating the quantitatively modified CDC assay

The above secondary standard (non‐inactivated version) was employed to create a set of calibrators at 10^8.5^, 10^7.5^, 10^6.5^, 10^5.5^, and 10^4.5^ IU/ml. These were incorporated into the CDC protocol with each assay iteration, to generate a linear equation for numeric quantification. Prior to implementation, however, the performance characteristics of this modified assay required validation. Many of these parameters remained unchanged from the original assay (i.e., accuracy, precision, analytic sensitivity, and analytic specificity), and we recapitulated these studies to verify explicitly the original CDC data (not shown).^22^ Importantly, though, our assay's quantitative modification required the additional validation of its dynamic range.

Accordingly, its LLOQ was determined according to the CLSI EP17‐A2,^36^ with validation specimens contrived from the WHO primary standard (see Section 2). The results of these experiments, along with bias analysis, are summarized in Table 1. Under both RMS and Westgard methods, the total observed error remained under an allowable limit of 0.5 down to 10^3.4^ IU/ml, for both the N1 and N2 primer/probe sets. This value was subsequently defined as the LLOQ; of note, it is only slightly higher than the assay's observed limit of detection with this protocol (10^3^, defined as the lowest value at which >95% of replicates generate a measurable Ct‐value, regardless of linearity). By contrast, the assay's upper limit of quantification (ULOQ) was set at the value of the highest calibrator (10^8.5^ IU/ml), given the lack of available validation materials above this concentration. With this dynamic range, specimens with a measured VL of 10^3.4^–10^8.5^ IU/ml (average of N1 and N2 calculations) were reported with the numeric value. Otherwise, they were considered *Detected, above/below the limit of quantification* (as applicable).

**TABLE 1:** Lower limit of quantification (LLOQ) validation.

	Nominal concentration (10^×^ IU/ml)	Calculated concentration, mean (SD)	Bias	TE‐1	TE‐2
N1					
	5.00	4.90 (0.12)	−0.10	0.34	0.34
	4.00	3.91 (0.13)	−0.09	0.34	0.36
	3.70	3.53 (0.13)	−0.12	0.37	0.35
	**3.40**	**3.23 (0.16)**	**−0.17**	**0.48**	**0.44**
	3.00	2.80 (0.27)	−0.20	0.75	0.78
N2					
	5.00	4.92 (0.10)	−0.08	0.28	0.28
	4.00	3.91 (0.10)	−0.09	0.28	0.28
	3.70	3.60 (0.15)	−0.10	0.40	0.42
	**3.40**	**3.28 (0.16)**	**−0.12**	**0.44**	**0.45**
	3.00	2.83 (0.22)	−0.17	0.61	0.64

*Note*: Viral loads were calculated, based on secondary calibrators, for various lots of low‐positive SARS‐COV‐2 specimens (derived from the WHO primary standard and negative clinical matrix). Summarized here—for both N1 and N2 primer/probe sets—are the mean, standard deviation, and bias of these calculations, relative to the nominal concentrations. For each viral concentration, the total error was calculated according to both RMA and Westgard methods. Based on a total allowable error of 0.5, 10^3.4^ IU/ml is considered the LLOQ for both N1 and N2.

Abbreviations: IU/ml, International Units/milliliter; SD, standard deviation; TE‐1, total error as calculated by Westgard Method; TE‐2, total error as calculated by root mean squared (RMS) method.

### 3.3 Applicability of secondary standard/calibrators to other platforms

**FIGURE 3:**
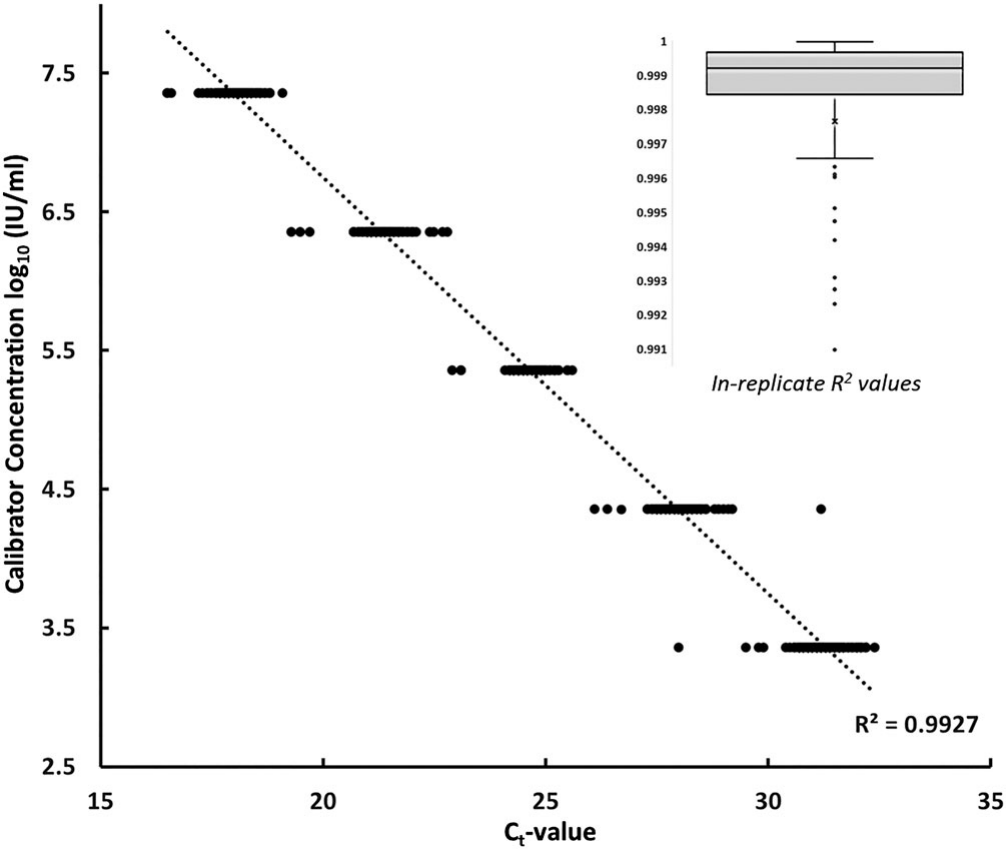
Linearity of calibrators on the Hologic RT‐qPCR assay. Serial dilutions of the contrived secondary standard were applied to this automated IVD assay for SARS‐CoV‐2. The C_t_‐value response is plotted here across these numerous experiments (*n* = 198), together with composite linear regression analysis across these replicates. Individual in‐replicate trendlines are not depicted, because of the large number of replicates, but consistently demonstrated high linearity. This range of in‐replicate *R* ^2^ values is summarized by the inset box‐and‐whispers plot.

With the above validation, the modified CDC assay met the criteria for clinical implementation. At the same time, this secondary standard could hold commensurate value for other SARS‐CoV‐2 assays to facilitate quantification, including high‐throughput commercial platforms. The calibrators were thus evaluated for a linear dose–response on an additional IVD assay, the fully automated Hologic Panther Fusion assay. Dilutions of the standard (at final concentrations of 10^3.6^–10^7.6^ IU/ml within the *specimen lysis tubes*) were applied to 198 independent replicates of this test, demonstrating extremely high linearity between viral concentration and C_t_‐value. In‐replicate linearity across this range demonstrated a median *R* ^2^ > 0.99, with a composite *R* ^2^ across all runs of 0.987 (Figure 3).

## 4 DISCUSSION

The massive scale of the SARS‐CoV‐2 pandemic, especially from 2020 into 2022, highlighted numerous challenges in the realm of diagnostic testing. These included key analytic issues, such as the differential performance of numerous assay options, the ability to compare results among these different tests, and the relationship between the indication for testing (e.g., symptomatic testing, asymptomatic screen, and test of cure) and the clinical interpretation of the result.^16^, ^38^, ^39^ At the same time, logistical challenges were an equally prominent factor in SARS‐CoV‐2 diagnostics. These included widespread unavailability (or suboptimal availability) of laboratory reagents, the role of commercially promulgated versus locally developed testing options, and the regulatory requirements that assay implementation.^19^, ^39^, ^40^ The issue of quantitative SARS‐CoV‐2 testing lies at the intersection of all the above.

Despite these challenges, the use of derivative‐of‐care materials to formulate a secondary standard allowed us to implement rigorous VLs. Such testing was available made locally in Spring 2021, albeit only when a quantitative result could impact clinical management (requiring approval by both specialist providers and laboratory leadership). Although this test no longer remains active clinically here—with the waning of the pandemic and accordant need—it could theoretically be reimplemented should the circumstances arise. Of note, however, we continue to utilize derivative‐of‐care calibrators for high‐throughput epidemiologic studies for SARS‐CoV‐2, research that does not require a clinical laboratory environment, per se, but still demands many of the same analytic principles and logistics. Recently published work has demonstrated advantages in cross‐laboratory precision with molecular calibration against primary WHO standard,^41^ and such practices could be further updated/expanded through traceable secondary standards.

Overall, the standard/calibrators described here demonstrate several key strengths, including the features delineated in Section 1 (IU traceability, clinical matrix specificity, native analyte structure, full extractability, broad concentration range, cost favorability, and large volumes). At the same time, we acknowledge several inherent limitations of this strategy. For instance, the pooled specimens were collected from a discrete period of time and would naturally reflect the predominant circulating strains. If the virus were to evolve in a manner that impacts the analytic sensitivity of the underlying RT‐qPCR assay, the calibrators would need to be updated to reflect an accurate VL (along with the assay itself).

Revalidation would likewise be necessary if the site of collection (nasopharyngeal versus BAL fluid) or collection media were changed. It is also important to note that, for VTM specimens, any VL represents the concentration of analyte within this external matrix. Naturally, this value is affected by the amount of VTM used to collect and, on a patient‐by‐patient basis, the vigor of the swabbing technique. However, these issues are universal to any SARS‐CoV‐2 NAT methodology and calibrator, and they are not specific to this particular scenario. Finally, although the realistic state of the virus within the calibrators offers certain advantages, we recognize that some laboratories might prefer not to employ viable pathogen. Therefore, we also replicated our characterization of the secondary standard assignment following heat inactivation, with negligible differences in the assigned concentration.

Of course, the COVID‐19 pandemic was extraordinary and some of the circumstances surrounding diagnostic testing for this virus may not repeat themselves in the immediate future. Nevertheless, we believe that our strategy for creating a secondary standard and calibrators may hold relevance for pathogens beyond SARS‐CoV‐2, whether for diagnostic or research motivations. For many viruses and fastidious bacteria (including intracellular bacteria), native and high‐concentration materials are more readily attainable from routine clinical practice than any commercial sources. As with SARS‐CoV‐2, these are the sorts of pathogens for which local in vitro culture (to generate high‐concentration stocks) is often not practicable. With nonstandard specimen types, moreover, commercial standards (when available) must first be diluted in the relevant matrix to create clinically realistic calibrators. From a global perspective, even if quantified microbial products are available from select providers, the associated costs are often substantial and distribution options may be limited. Repurposing derivative‐of‐care materials could represent a financially viable alternative to ensure rigorous quantification in such locations. If a WHO‐promulgated primary standard is not available for a given pathogen, an alternate (non‐IU) based material could be employed, albeit one that is available as broadly as possible and with detailed documentation, including the method of initial concentration assignment.

In summary, we report a novel approach for generating an IU‐traceable secondary standard for SARS‐CoV‐2, even during a time of no viable commercial options. This standard was utilized to validate a quantitative VL assay for specialized clinical circumstances, demonstrating how creative calibration strategies can facilitate high‐quality testing despite resource limitations.

## AUTHOR CONTRIBUTIONS

**Lili Tao:** Data curation; formal analysis; methodology; writing—review and editing. **Allison Chan:** Formal analysis; writing—original draft; writing—review and editing. **Alex Maris:** Formal analysis; writing—review and editing. **Jonathan E. Schmitz:** Conceptualization; data curation; formal analysis; funding acquisition; investigation; methodology; project administration; resources; validation; visualization; writing—original draft; writing—review and editing.

## CONFLICT OF INTEREST STATEMENT

No conflict of interest was declared.

### PEER REVIEW

The peer review history for this article is available at https://www.webofscience.com/api/gateway/wos/peer-review/10.1111/irv.13207.

## ETHICS STATEMENT

Prior to pooling the secondary standard, specimens were stripped of protected health information and permanently de‐identified from the source patients. This work was performed in accordance with protocols approved by the Vanderbilt Institutional Review Board.

## Data Availability

The data that support the findings of this study are available from the corresponding author upon reasonable request.
